# Comparative Study on the Therapeutic Potential of Neurally Differentiated Stem Cells in a Mouse Model of Multiple Sclerosis

**DOI:** 10.1371/journal.pone.0035093

**Published:** 2012-04-13

**Authors:** Natalie L. Payne, Guizhi Sun, Daniella Herszfeld, Pollyanna A. Tat-Goh, Paul J. Verma, Helena C. Parkington, Harold A. Coleman, Mary A. Tonta, Christopher Siatskas, Claude C. A. Bernard

**Affiliations:** 1 Monash Immunology and Stem Cell Laboratories, Monash University, Clayton, Victoria, Australia; 2 Centre for Reproduction and Development, Monash Institute of Medical Research, Monash University, Clayton, Victoria, Australia; 3 Department of Physiology, Monash University, Clayton, Victoria, Australia; Dana-Farber Cancer Institute, United States of America

## Abstract

**Background:**

Transplantation of neural stem cells (NSCs) is a promising novel approach to the treatment of neuroinflammatory diseases such as multiple sclerosis (MS). NSCs can be derived from primary central nervous system (CNS) tissue or obtained by neural differentiation of embryonic stem (ES) cells, the latter having the advantage of readily providing an unlimited number of cells for therapeutic purposes. Using a mouse model of MS, we evaluated the therapeutic potential of NSCs derived from ES cells by two different neural differentiation protocols that utilized adherent culture conditions and compared their effect to primary NSCs derived from the subventricular zone (SVZ).

**Methodology/Principal Findings:**

The proliferation and secretion of pro-inflammatory cytokines by antigen-stimulated splenocytes was reduced in the presence of SVZ-NSCs, while ES cell-derived NSCs exerted differential immunosuppressive effects. Surprisingly, intravenously injected NSCs displayed no significant therapeutic impact on clinical and pathological disease outcomes in mice with experimental autoimmune encephalomyelitis (EAE) induced by recombinant myelin oligodendrocyte glycoprotein, independent of the cell source. Studies tracking the biodistribution of transplanted ES cell-derived NSCs revealed that these cells were unable to traffic to the CNS or peripheral lymphoid tissues, consistent with the lack of cell surface homing molecules. Attenuation of peripheral immune responses could only be achieved through multiple high doses of NSCs administered intraperitoneally, which led to some neuroprotective effects within the CNS.

**Conclusion/Significance:**

Systemic transplantation of these NSCs does not have a major influence on the clinical course of rMOG-induced EAE. Improving the efficiency at which NSCs home to inflammatory sites may enhance their therapeutic potential in this model of CNS autoimmunity.

## Introduction

Therapeutic transplantation of neural stem/precursor cells (NSCs) is currently being investigated as a novel treatment strategy for multiple sclerosis (MS) and other neurodegenerative diseases [1]. Although originally based on the concept of cell replacement, evidence emanating from studies in experimental autoimmune encephalomyelitis (EAE), an animal model which mimics many features of MS, has revealed little evidence for the remyelinating capacity of transplanted NSCs. Rather, the improved clinical outcome appears to result from bystander immunomodulatory and neuroprotective effects, exerted by NSCs in response to signals from the surrounding microenvironment, which collectively dampen inflammation, inhibit glial scar formation and enhance neurogenesis [2], [3], [4], [5]. Importantly for the treatment of multifocal diseases such as MS, systemically injected NSCs have been reported to not only regulate immune responses in peripheral lymphoid tissues [5], [6], [7], but also migrate across the blood brain barrier into the CNS parenchyma [4], [5]. Although the molecular mechanisms governing NSC homing from the vasculature to sites of CNS pathology remain undefined, expression of molecules important in leukocyte trafficking are thought to play an important role [8].

Despite these encouraging pre-clinical studies, several outstanding issues surrounding the clinical translation of NSC-based therapies still remain unresolved, including the optimal dose, route of transplantation and cell source. EAE transplantation studies have predominantly focused on primary mouse NSCs derived from neurogenic regions of the brain. While human fetal NSCs have already been used in clinical trials [9], these cells may not represent a suitable cell source for large scale therapeutic transplantation [10]. Alternatively, embryonic stem (ES) cells, or more recently developed induced pluripotent stem cells, may provide an unlimited source of NSCs for cell-based therapies. The protocols used for neural differentiation of ES cells have traditionally involved propagation of neurospheres [11], which are highly heterogeneous floating cell clusters comprised of a small number of NSCs in addition to progenitors with limited differentiation potential. In order to overcome problems associated with the heterogeneity of neurosphere cultures, a protocol to derive and expand NSCs in the presence of basic fibroblast growth factor (bFGF) and epidermal growth factor (EGF) via niche independent adherent monocultures has been developed, allowing large scale production of uniform, symmetrically renewing NSCs with a tri-lineage differentiation potential [12], [13], [14].

The ability of ES cell-derived NSCs to undergo migration and differentiation when transplanted directly into the developing or adult brain has been described in numerous studies [13], [15], [16], [17], however much less is known about their therapeutic efficacy in neuroinflammatory disease models such as EAE, particularly when delivered by a systemic route. The current study sought to evaluate the therapeutic effect of systemically administered NSCs derived from ES cells in a chronic progressive murine model of MS induced by recombinant myelin oligodendrocyte glycoprotein (rMOG). We chose to focus on NSCs derived by two different ES cell differentiation protocols that utilize adherent culture conditions. 46C-NS cells are a homogenous, symmetrically dividing NSC population derived from ES cells in the presence of bFGF and EGF, while GS-N cells are comprised of a heterogeneous population of progenitor cells similar in composition to neurospheres. As a further comparison, we have utilized NSCs derived from the subventricular zone of adult mice (SVZ-NSCs), which are expanded using the free floating neurosphere system. We show that only GS-N cells and SVZ-NSCs can effectively suppress both T-cell proliferative responses and pro-inflammatory cytokine production *in vitro*, however the homing efficiency of systemically administered cells limits the efficacy NSC transplantation in this model of CNS autoimmunity.

## Results

### Characterization of neurally differentiated ES cells

**Figure 1:**
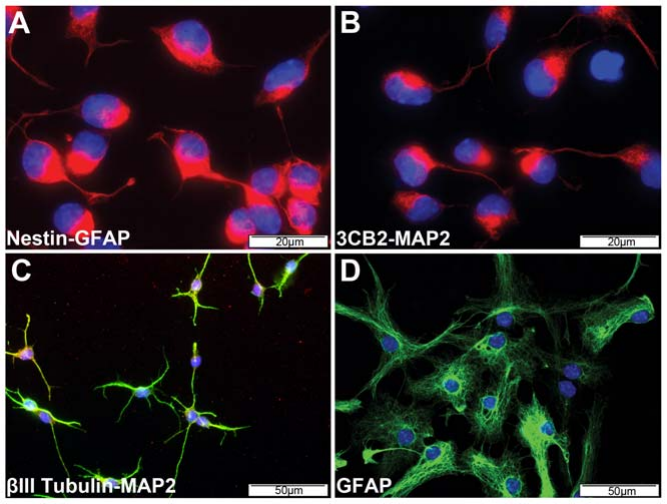
Characterization of 46C-NS cells. (A–C) Immunofluorescence staining showed 46C-NS cells expressed nestin, red (A) and 3CB2, red (B) but not GFAP, green (A) or MAP2, green (B). (**D–E**) Following neuronal or astrocytic differentiation, 46C-NS cells expressed βIII-Tubulin, red and MAP2, green (C) or GFAP (D), respectively.

Previous reports have described the differentiation of mouse ES cells into uniform and symmetrically dividing NSCs using serum-free adherent monocultures [12], [13]. 46C-NS cells are one well characterized NSC line derived from *Sox1*-green fluorescent protein (GFP) knock in reporter ES cells [12]. Confirming results presented in earlier studies, we found that 46C-NS cells uniformly expressed nestin (Fig. 1A) and the radial glia marker 3CB2 (Fig. 1B), which recognizes three isoforms of vimentin [18], but not the astrocytic marker glial fibrillary acidic protein (GFAP) (Fig. 1A) or neuronal marker microtubule-associated protein-2 (MAP2) (Fig. 1B). Under appropriate differentiation conditions 46C-NS cells demonstrated a neuronal and astrocytic differentiation potential, as determined by positive staining for βIII-tubulin and MAP2 (Fig. 1C) and GFAP (Fig. 1D).

**Figure 2:**
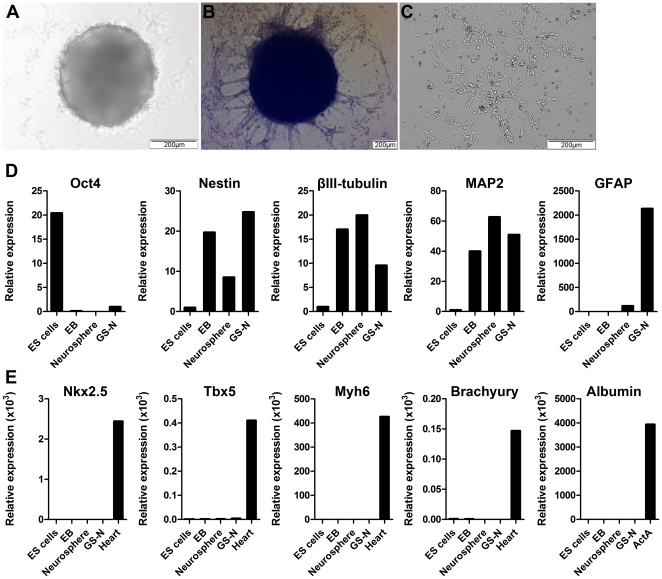
Gene expression profile of GS-N cells. (A) ES cells aggregated to form round floating EBs. (B) Day 4 neurospheres with neural projections generated from EBs. (C) Day 4 GS-N cells generated by dissociation of EBs. (D–E) Quantitative real time PCR analysis of the expression of neuroectodermal genes (D) and mesodermal and endodermal genes (E) by ES cells, day 7 EBs, day 4 neurospheres and day 4 GS-N cells. cDNA from embryonic heart tissue and Activin A (ActA) treated EBs served as positive controls.

We next developed a protocol for neural differentiation of ES cells, with the aim of generating a heterogeneous population of NSCs and progenitor cells that could be grown under adherent culture conditions, as opposed to employing the free-floating neurosphere culture system. Embryoid bodies (EBs) were first derived from ES cells via the spin EB method [19] and then cultured in non-adherent 96-well plates for 7 days (Fig. 2A). These EBs could be used to generate neurospheres (Fig. 2B) or could be mechanically dissociated and re-plated onto poly-L-ornithine/laminin coated plates for 4 days to generate adherent cells termed GS-N cells (Fig. 2C). In order to verify neuroectodermal specification, the gene expression profile of these cells was examined by quantitative real time PCR. The transcription factor Oct4, a marker of pluripotency, was downregulated in day 7 EBs, day 4 neurospheres and day 4 GS-N cells compared to ES cells (Fig. 2D). In contrast, the neuroectodermal marker nestin was upregulated in EBs, GS-N cells and to a lesser extent in neurospheres. Interestingly, while expression of the neuronal markers β-III tubulin and MAP2 also increased during neural induction, only GS-N cells showed highly upregulated expression of GFAP. Transcripts for 2′,3′-cyclic nucleotide 3′-phosphodiesterase, an enzyme almost exclusively expressed by myelinating cells [20], could also be detected in GS-N cells by RT-PCR (data not shown), while expression of genes marking the mesodermal and endodermal germ lineages were not detected (Fig. 2E).

**Figure 3:**
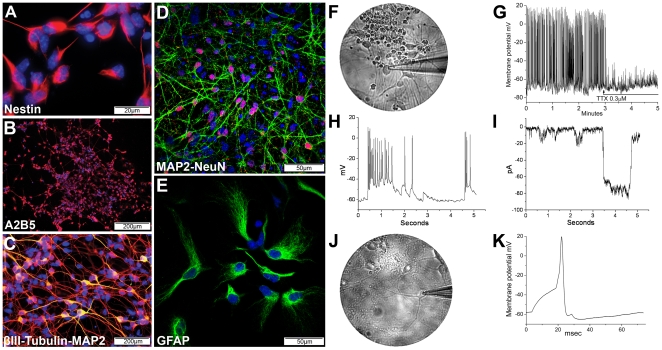
Differentiation potential of GS-N cells. (A–C) Immunofluorescence staining showed GS-N cells expressed nestin, red (A), A2B5, red (B) and βIII-Tubulin, red and MAP2, green (C). (D–E) Following neuronal or astrocytic differentiation, GS-N cells expressed MAP2, green and NeuN, red (D) or GFAP (E), respectively. (F–K) Electrophysiological assessment of GS-N cell-derived neurons. Neurons in high density (F) fired spontaneous action potentials (APs), which were blocked by tetrodotoxin (TTX) (G). The depolarization giving rise to the APs (H) was underpinned by an inward current (I). Sparsely growing neurons (J) did not display spontaneous activity but APs could be evoked by depolarising current steps (K).

Immunofluorescence further confirmed that the GS-N cells were comprised of a heterogeneous population of neural progenitors and lineage restricted cells expressing nestin (Fig. 3A), the oligodendrocyte progenitor marker A2B5 (Fig. 3B) and βIII-tubulin and MAP2 (Fig. 3C). To promote neuronal differentiation, GS-N cells were cultured for an additional two weeks in the absence of growth factors. Under these conditions, increased numbers of mature MAP2^+^NeuN/Fox-3^+^ neuronal cells were generated (Fig. 3D). Alternatively, when GS-N cells were exposed to serum to promote astrocytic differentiation, substantial numbers of GS-N cells adopted an astrocytic morphology and expressed GFAP (Fig.3E). Quantitative analyses revealed that these differentiated cultures contained 69.28%±6.14 GFAP^+^ cells.

To demonstrate that functional neural cell types could be generated using our ES cell differentiation protocol, the electrophysiological properties of neuronally differentiated GS-N cells were assessed by making patch-clamp recordings in whole-cell, current clamp mode. These cells had resting membrane potentials of −63±2 mV, input resistances of 1.4±0.3 GΩ, and input capacitances of 25±2 pF. The neuronal cells that formed dense networks with many interconnecting processes (Fig. 3F) were found to fire action potentials (APs) spontaneously (Fig. 3G, H), with amplitudes of 81±7 mV and width at half amplitude of 2.7±0.8 ms. Spontaneous APs occurred in 69% of cells and were abolished by the Na^+^ channel blocker tetrodotoxin (TTX) (Fig. 3G). APs occurred due to the development of spontaneous depolarisations, which took the membrane potential beyond the threshold for the opening of TTX-sensitive voltage-gated Na^+^ channels (Fig. 3G, H). Study of these spontaneous events in voltage clamp, and in the presence of TTX to prevent APs, revealed the underlying inward currents (Fig. 3I). Notably, cells with limited network connections to other cells (Fig. 3J) generated smaller and less frequent depolarisations, and hence fired few spontaneous APs. Although no APs occurred spontaneously in 31% of cells, APs could be evoked by depolarising current steps and these had characteristics that were indistinguishable from APs that occurred spontaneously (Fig. 3K). Collectively, these results demonstrate that our differentiation protocol could yield a heterogeneous population of adherently growing cells, which expressed markers representative of all three neural lineages and could be further differentiated into functional neurons.

### GS-N cells and 46C-NS cells exert differential effects on T-cells *in vitro*

**Figure 4:**
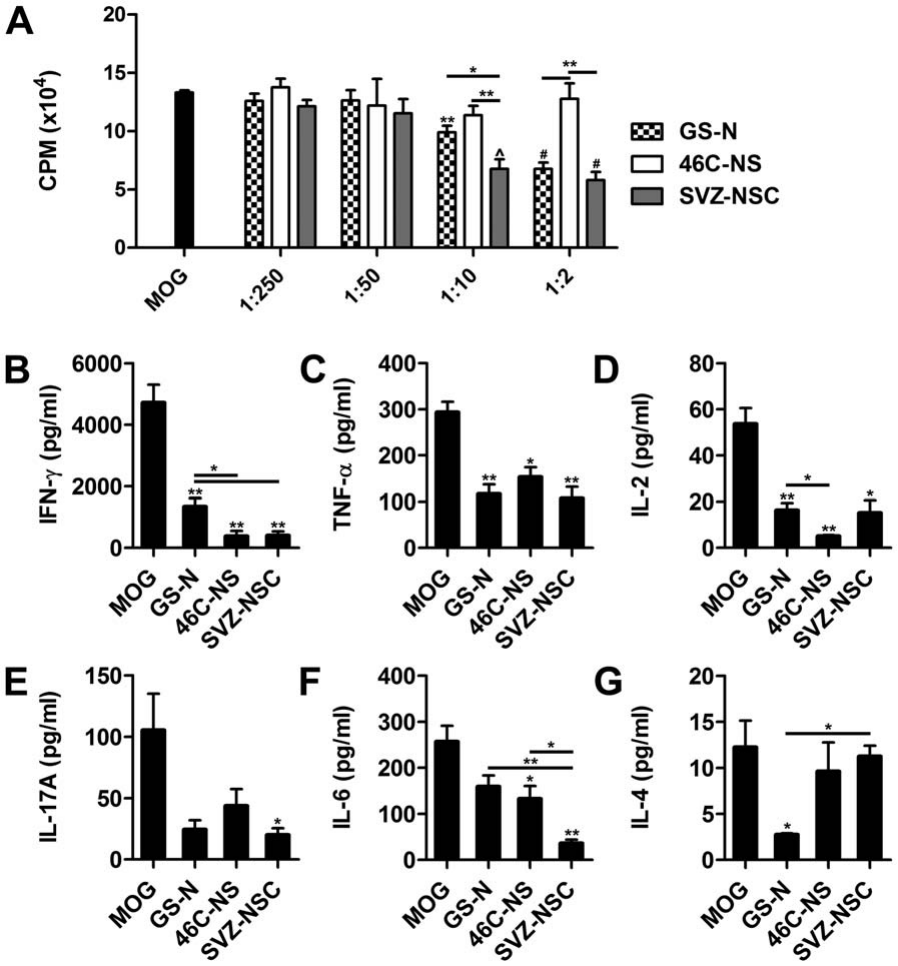
Suppression of *in vitro* MOG-specific T-cell responses. (A) Fixed numbers of splenocytes from 2D2 mice stimulated with MOG_35–55_ were cultured in the presence of varying numers of GS-N cells, 46C-NS cells or SVZ-NSCs (expressed as NSC: splenocyte ratio). Proliferative responses were measured by ^3^H-thymidine incorporation and expressed as the mean counts per minute (CPM). (B–G) Supernatants were collected from co-cultures after 48 hrs and the level of pro-inflammatory cytokines was quantified by cytometric bead array. Data represent the mean ± SEM (n = 4 mice). *P<0.05, **P<0.01, ∧P<0.005, ^#^P<0.001 vs MOG_35–55_ stimulated splenocytes unless otherwise indicated.

Attenuation of inflammatory processes within the CNS and peripheral lymphoid tissues is an important mechanism by which NSCs mediate clinical recovery in EAE [5], [6], [7], [21]. To directly compare their immunosuppressive potential in an antigen-specific setting, varying numbers of GS-N cells or 46C-NS cells were co-cultured with fixed numbers of splenocytes from 2D2 mice, which express the transgenic Vα3.2/Vβ11 T-cell receptor specific for the encephalitogenic myelin oligodendrocyte glycoprotein (MOG)_35–55_ peptide [22]. We also simultaneously assessed the immunosuppressive properties of SVZ-NSCs propagated by the neurosphere system, as this cell source and culture method is commonly employed for studying NSC transplantation in EAE and other CNS disease models. GS-N cells suppressed T-cell proliferative responses to MOG_35–55_ in a dose-dependent manner, with significant inhibition observed at stem cell∶splenocyte ratios of 1∶2 and 1∶10 (Fig. 4A). The immunosuppressive capacity of GS-N cells was comparable to SVZ-NSCs at high cell numbers (ratio 1∶2, 45.2%±4.3 vs 53.1%±5.7 T-cell inhibition, respectively), however GS-N cells were significantly less efficient at inhibiting T-cell proliferation compared to SVZ-NSCs at lower cell numbers (ratio 1∶10, 25.5%±4.6 vs 52.6%±7.8 T-cell inhibition, respectively). Nevertheless, the immunosuppressive capacity of GS-N cell was significantly more potent than 46C-NS cells, which exerted no inhibitory effect on T-cell proliferative responses at any of the ratios tested.

To analyze the effect of GS-N cells, 46C-NS cells and SVZ-NSCs on cytokine production by MOG_35–55_-stimulated splenocytes, supernatant was collected from co-cultures in which maximal inhibition of T-cell proliferation was observed (ratio 1∶2). The levels of key cytokines known to play a role in EAE pathogenesis [23] were then quantified by cytometric bead array. Production of the pro-inflammatory cytokines interferon (IFN)-γ, tumor necrosis factor (TNF)-α, interleukin (IL)-2, IL-17A and IL-6 was reduced when MOG_35–55_-stimulated splenocytes were co-cultured with GS-N cells or SVZ-NSCs (Figs. 4B–F). Unexpectedly, 46C-NS cells also inhibited the secretion of these cytokines, despite the fact that T-cell proliferative responses were unaffected. Moreover, IL-4 production by stimulated splenocytes was reduced in the presence of GS-N cells but not 46C-NS cells or SVZ-NSCs (Fig. 4G), suggesting that different mechanisms of action may underlie the immunosuppressive properties of these cells. Taken together, SVZ-NSCs appear to exhibit the most potent suppressive capacity *in vitro*, however all three cell types have the capacity to reduce the secretion of cytokines known to mediate inflammatory processes in EAE.

### Intravenously injected NSCs do not attenuate rMOG-EAE

**Figure 5:**
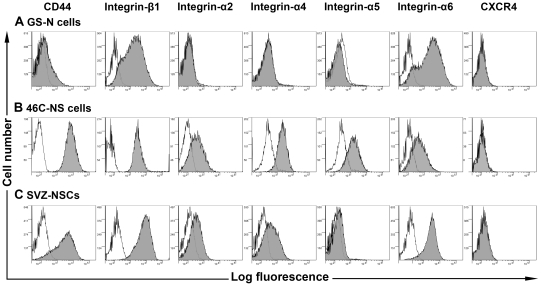
Effect of intravenous NSC transplantation on the clinical course of EAE. (A) C57Bl/6 mice were immunized with recombinant myelin oligodendrocyte glycoprotein and treated with intravenous injections of GS-N cells, 46C-NS cells or SVZ-NSCs on days 8, 10 and 12 post immunization. (B) Proliferative response of splenic T-cells from cell-treated mice and controls to rMOG and non-specific mitogens expressed as the mean counts per minute (CPM). Data are expressed as the mean ± SEM (n = 5–7 mice). (C) Decreasing amounts of DNA from GFP^+^ GS-N cells were titrated with DNA extracted from EAE mice and the minimum amount of GFP^+^ DNA detectable was determined by PCR using primers specific for GFP. (D) GFP^+^ GS-N cells or fibroblasts were injected intravenously into EAE mice at 12 days post disease induction and PCR was used to detect GFP^+^ cells. PCR reactions performed without DNA served as a negative control and DNA from C57Bl/6-GFP chimeric mice served as a positive control.

We next evaluated the therapeutic efficacy of GS-N cells, 46C-NS cells and SVZ-NSCs in chronic-progressive EAE induced by rMOG, a model in which both T- and B-cells contribute pathogenetically [24], [25]. Three intravenous (i.v.) injections of 1×10^6^ cells were administered to EAE mice 8, 10 and 12 days post disease induction, at a time when the blood brain barrier is open. Surprisingly, none of the NSC-treated groups displayed a delay in the onset of clinical signs compared to PBS-injected controls (GS-N cells: 13.25±0.49, 46C-NS cells: 13.14±0.77, SVZ-NSC: 15.57±2.60, PBS: 14.29±0.57). Furthermore, no difference in the mean daily clinical score (Fig. 5A) or other disease metrics, such as the cumulative disease score or maximum clinical score, was observed. Spleens were then dissected on day 31 post disease induction in order to assess the impact of NSC transplantation on peripheral T-cell responses. Consistent with the clinical data, re-call proliferation assays showed that the proliferative response of splenocytes to MOG_35–55_ and non-specific mitogens was similar between the three NSC-treated groups and controls (Fig. 5B).

### Inefficient recruitment to sites of inflammation reduces the efficacy of GS-N cell transplantation

The absence of a beneficial effect in EAE mice following NSC therapy, despite the ability to suppress immune responses *in vitro*, could be due to the inefficient recruitment of transplanted cells to peripheral lymphoid tissues and areas of neuroinflammation. This may be a consequence of passive arrest and apoptosis within the pulmonary capillaries, as demonstrated previously for NSCs [26], and/or the lack of appropriate surface receptor expression and inability to actively home to inflamed tissues. To investigate this, we derived GS-N cells from GFP^+^ ES cells in order to track their *in vivo* biodistribution by PCR. The detection limit for this assay, determined by titrating varying amounts of DNA from GFP^+^ GS-N cells with DNA obtained from EAE mice, was found to be 1 ng of GFP^+^ DNA, which corresponded to 150 GFP^+^ cells (Fig. 5C). At day 12 post disease induction (the onset of clinical disease), GFP^+^ GS-N cells or mouse embryonic fibroblasts (MEFs) as a control cell type, were injected i.v. into EAE mice and various tissues were collected for analysis 24 hrs later. Transplanted GS-N cells could not be detected within the CNS or secondary lymphoid tissues, and only in one instance could the presence of GFP^+^ cells be identified within the lungs of EAE mice (data not shown). Analysis of the biodistribution of GFP^+^ GS-N cells during the chronic stage of disease (23 days post injection) revealed that GFP^+^ cells were only present in the kidney but not in the CNS, secondary lymphoid organs or lungs (Fig. 5D). These results suggest that GS-N cells were unable to migrate to and engraft at inflammatory sites, thus contributing to their lack of efficacy following i.v. transplantation into EAE mice.

**Figure 6:**
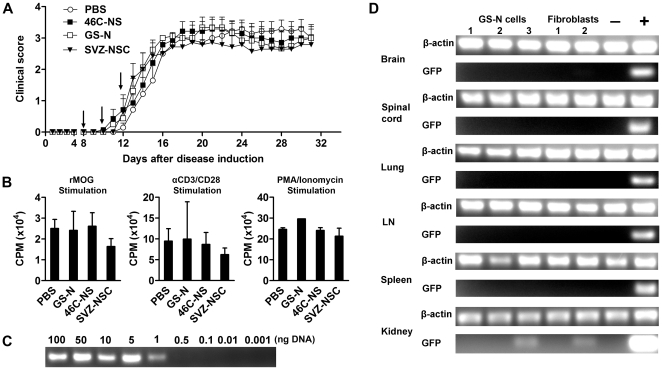
Cell surface expression of homing molecules. Flow cytometric analysis was used to examine cell surface expression of homing molecules by GS-N cells (A), 46C-NS cells (B) and SVZ-NSCs (C). Representative histograms are shown in grey and respective isotype controls are indicated by the black curve.

The molecular mechanisms governing the recruitment and migration of NSCs from the vasculature to inflamed sites are not yet well defined, but are likely to involve adhesion molecules and chemokine receptors that are important for leukocyte homing, such as very late antigen-4 (VLA-4; integrin-α4β1) [8]. Flow cytometric analysis showed that GS-N cells lacked surface expression of several adhesion molecules, namely the hyaluronic acid receptor CD44, integrin-α2, integrin-α4 and integrin-α5 whereas both integrin-β1 and integrin-α6 were expressed (Fig. 6A). In contrast, 46C-NS cells constitutively expressed varying levels of CD44 and all five integrins (Fig. 6B), indicating that these cells may have a greater capacity for adhesion and migration across inflamed endothelium compared to GS-N cells. SVZ-NSCs expressed CD44, integrin-β1, integrin-α2, integrin-α4 and integrin-α6 but lacked expression of integrin-α5 (Fig. 6C). Surprisingly, the SDF-1α receptor CXCR4, which has been implicated in targeted recruitment of cells to the CNS [27] as well as mediating their firm adhesion to brain endothelium [28], was not expressed on the surface of any of our three NSC types. Thus, the absence of homing molecules may have contributed to the limited therapeutic potential of NSCs in rMOG-induced EAE.

### Intraperitoneal injections of GS-N cells modulates EAE

**Figure 7:**
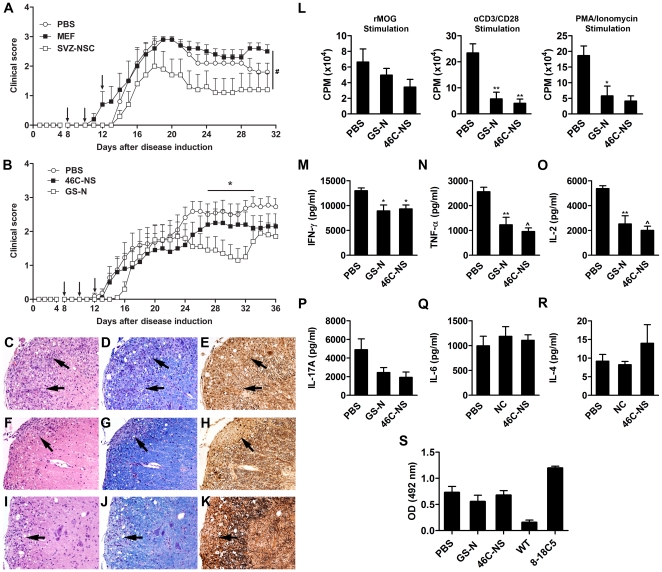
Effect of intraperitoneal NSC transplantation on the clinical course of EAE. C57Bl/6 mice were immunized with recombinant myelin oligodendrocyte glycoprotein and treated with intraperitoneal injections of NSCs on days 8, 10 and 12 post immunization. (A) EAE mice received 1×10^6^ SVZ-NSCs or MEFs. Data are expressed as the mean clinical score ± SEM (n = 5 mice). (B) EAE mice received 5×10^6^ GS-N cells or 46C-NS cells. Data are expressed as the mean clinical score ± SEM (n = 9–10 mice). Arrows indicate days of injection. (C–K) Representative spinal cord sections of PBS control mice (C–E), GS-N cell-treated mice (F–H), or 46C-NS cell-treated mice (I–K) were stained with haematoxylin and eosin, luxol fast blue and Bielschowsky silver stain to detect inflammation, demyelination and axonal damage, respectively. Magnification ×400. (L) Proliferative response of splenic T-cells from cell-treated mice and controls to rMOG and non-specific mitogens expressed as the mean counts per minute (CPM). (M–R) Supernatant from rMOG-stimulated splenocyte cultures were collected after 48 hrs and cytokines were quantified by cytometric bead array. (S) Serum rMOG-specific antibody response from NSC-treated mice and controls. Data are expressed as the mean ± SEM (n = 9–10 mice). *P<0.05, **P<0.01, ∧P<0.001 versus PBS controls, ^#^P<0.05.

Previous studies have shown that intraperitoneally (i.p.) delivered mesenchymal stem cells are capable of attenuating EAE [29], [30], [31], providing insight into peripheral immunomodulatory mechanisms that mediate their therapeutic effect whilst avoiding passive entrapment of transplanted cells within the pulmonary capillary bed. Subcutaneously injected NSCs can inhibit T-cell priming within the lymph nodes of EAE mice when transplanted prior to the onset of clinical signs [32], however the impact of i.p. injected NSCs on the course of EAE has not yet been determined. To test the feasibility of this delivery route we initially transplanted SVZ-NSCs, as these cells demonstrated the most potent immunosuppressive properties *in vitro*, as well as MEFs as a control cell type. When 1×10^6^ cells were administered i.p. at days 8, 10 and 12 post disease induction, mice receiving SVZ-NSCs displayed a reduced mean daily clinical score compared to PBS-injected controls and a significant reduction compared to MEF-treated mice (Fig. 7A). On the basis of these results, we chose to administer a higher dose (5×10^6^ cells per injection) to test the efficacy of i.p. injected GS-N cells and 46C-NS cells. In this experimental setting, GS-N cell-treated mice showed a significant decrease in disease severity during the chronic stage (days 27–33) compared with PBS-injected controls (Fig. 7B). Furthermore, GS-N cell treatment led to a reduction in the cumulative disease score as well as a significant reduction in the maximum clinical score (p = 0.0249, Table 1). Despite the high number of cells transplanted, 46C-NS cells failed to show any significant impact on the disease course.

**Table 1:** Clinical and pathological outcome of EAE mice.

	PBS control	GS-N cells	46C-NS cells
Disease incidence	11/11	9/10	8/10
Day disease onset (range)	19.64±1.75 (12–32)	22.40±2.53 (15–36)	22.20±2.69 (13–36)
Maximum score	3.09±0.18	2.45±0.32*	2.50±0.42
Cumulative score	50.50±6.98	32.50±7.41	40.02±7.70
Inflammatory score	2.41±0.33	1.65±0.39	1.35±0.29*
Demyelinating score	2.36±0.34	1.50±0.37	1.40±0.31
Axonal loss score	3.00±0.36	1.50±0.38**	1.95±0.42

Data are expressed as mean ± SEM.

* p<0.05,

** p<0.01 vs PBS.

Histopathological evaluation of haematoxylin and eosin stained spinal cord sections from PBS-treated mice showed typical extensive mononuclear inflammatory cell infiltration (Fig. 7C). Luxol fast blue (Fig. 7D) and Bielschowsky silver staining (Fig. 7E) also showed marked myelin loss and axonal injury, respectively. In comparison, GS-N cell-treated animals displayed reduced inflammation (Fig. 7F) with an associated preservation of the myelin architecture (Fig. 7G) and significantly reduced axonal damage (Fig. 7H, Table 1). Interestingly and despite the fact that 46C-NS cell-treatment did not alter the clinical course of disease, inflammatory infiltrates in the CNS of these mice were significantly reduced (Fig. 7I, Table 1). A trend towards reduced demyelination and axonal damage was also observed (Fig. 7J, K).

### Transplanted GS-N cells and 46C-NS cells suppress peripheral T-cell responses

To determine whether the reduction in CNS pathology of NSC-treated mice was due to suppression of peripheral immune responses, we first performed re-call proliferation assays to assess the *ex vivo* proliferative response of splenic T-cells to specific and non-specific mitogens. Although no significant difference in rMOG-specific T-cell responses was observed, there was a significant decrease in T-cell proliferation when splenocytes from GS-N and 46C-NS cell-treated mice were re-stimulated with anti-CD3/CD28 (GS-N cells: p = 0.0035; 46C-NS cells: p = 0.0011 vs PBS) or phorbol myristate acetate and ionomycin (GS-N cells: p = 0.0171; 46C-NS cells: p = 0.0035 vs PBS; Fig. 7L). Quantification of cytokine levels in anti-CD3/CD28 stimulated cultures showed that splenocytes from GS-N cell-treated mice produced significantly less IFN-γ, TNF-α and IL-2 (Fig. 7M–O) and reduced levels of IL-17A (Fig. 7P). Likewise, the level of these pro-inflammatory cytokines was also reduced for mice receiving 46C-NS cells. We further analyzed the effect of GS-N cells and 46C-NS cells on autoantibody production, however no significant difference in serum levels of rMOG-specific antibodies was found (Fig. 7S). Taken together, these results suggest that high numbers of GS-N cells or 46C-NS cells could suppress peripheral T-cell responses in a non-specific manner, leading to some neuroprotective effects within the CNS.

## Discussion

*Ex vivo* expanded NSCs derived from neurogenic regions of the brain possess an inherent therapeutic plasticity [33]. Indeed, clinical recovery has been induced by NSC transplantation in experimental models of traumatic brain injury, stroke, Parkinson's disease, Huntington's disease, spinal cord injury and MS. The mechanisms which mediate amelioration of EAE occur predominantly through bystander immunomodulatory and neuroprotective effects exerted by NSCs, rather than differentiation and functional integration in the CNS [34]. A critical issue for the successful clinical application of ES cells in CNS diseases is the ability to efficiently generate purified NSCs with this same therapeutic plasticity.

Numerous methods involving manipulation of the extrinsic culture environment, via the addition of various growth factors and modulation of signaling pathways, to induce neural specification of mouse and human ES cells have been described [35]. Although NSCs have been traditionally cultured as neurospheres, a more recent method has employed niche independent adherent culture conditions to generate uniform and symmetrically dividing NSCs [13], thereby avoiding the heterogeneity that develops within the neurosphere structure [36]. In the current investigation, we confirmed some of the phenotypic properties and the differentiation potential of 46C-NS cells described previously [13], [14]. Utilizing our own ES cell differentiation protocol, we generated a heterogeneous population of adherently cultured progenitor cells that expressed markers representative of all three neural lineages. Our electrophysiological assessment of GS-N cells undergoing neuronal differentiation confirmed that functional neural cell types could indeed be generated from this protocol. The characteristics of these neurons were similar to those reported for neurons in brain slices [37], in freshly isolated neurons [38], in neurons derived from human embryonic stem cells [39] and in neurons reprogrammed from fibroblasts [40]. Signaling networks are known to be critical for normal functioning of the nervous system [41]. Interestingly, we demonstrate here that the signaling networks formed by neuronally differentiated GS-N cells were also important for their functional properties, as action potentials occurred spontaneously when the density of neurons was higher and rarely occurred in neurons that were not close to each other.

Transplanted NSCs exert their therapeutic effect in part via non-specific immunosuppression [7], [21], which led us to compare the impact of GS-N cells and 46C-NS cells on *in vitro* T-cell proliferative responses as a prelude to transplantation studies. The ability of GS-N cells to inhibit T-cell proliferation was similar to that of SVZ-NSCs propagated via the neurosphere system at high cell numbers, whereas 46C-NS cells had remarkably little effect. The individual cell types within the heterogeneous neurosphere structure that account for the immunosuppressive properties of NSCs, as demonstrated by SVZ-NSCs in this study and by others [2], [7], [42], have not been extensively analyzed. Yet, the differentiation potential can influence the immunosuppressive properties of neural cell types, as astrocytes but not neurons are capable of suppressing T-cell responses *in vitro* [2], [43]. Furthermore, some adherent NSC cultures have a higher neuronal potential [44], which may limit their immunosuppressive capacity. An extensive analysis of methods for neural differentiation of ES cells and how these may influence the functional properties of NSCs was beyond the scope of this study. Thus, we have instead focused on a comparison of NSCs derived from ES cells by two different neural differentiation protocols that utilize adherent culture conditions, resulting in either a symmetrically dividing population (46C-NS cells) or heterogeneous population more reminiscent of neurospheres (GS-N cells). Whilst the uniformity of NSC cultures such as 46C-NS cells may be desirable, for instance to reduce experimental variability, it is interesting to note that in current setting, only heterogeneous NSC cultures were capable of suppressing T-cell proliferation. Nevertheless, secretion of pro-inflammatory cytokines IFN-γ, IL-2, TNF-α and IL-17 was reduced in the presence of GS-N cells and 46C-NS cells. Moreover, 46C-NS cells did not suppress the production of IL-4, which is associated with disease remission in EAE. Thus, ES cell-derived NSCs in general may have the potential to modify the cytokine secretion profile of immune cells, which could certainly exert a major influence on clinical outcomes in EAE [45] and possibly other neuroinflammatory conditions.

Systemically delivered primary NSCs have been shown to traffic to the CNS not only in EAE [4], [5], [46], [47], but also in mouse models of stroke [48] and Huntington's disease [49]. While the survival, migration and integration of neural differentiated ES cells injected directly into the CNS has been reported during development [15] and under pathological conditions [50], [51], [52], there is currently a paucity of information regarding their capacity to respond to tissue signals when delivered systemically. This study was therefore performed to assess whether neural differentiated ES cells could produce the same therapeutic effect that has been reported for NSCs derived from CNS tissue. We were unable to find any beneficial effect on the clinical course of disease after multiple i.v. injections of GS-N cells or 46C-NS cells were administered to mice with rMOG-induced EAE. Moreover, SVZ-NSCs did not attenuate EAE severity, which is in contrast to previous studies, in which substantial clinical recovery could be induced after a single i.v. injection of primary NSCs, irrespective of whether the cells were delivered before [4], [5], [7] or after [4], [5], [46], [47] disease onset. The influence of GS-N cells, 46C-NS cells and SVZ-NSCs on EAE induced by MOG_35–55_ is currently under investigation.

The reasons for these discrepancies are not readily apparent but could possibly be due to the low numbers of transplanted NSCs reaching inflammatory sites. Tracking the fate of GFP^+^ GS-N cells administered i.v. to EAE mice by PCR revealed that cells could only be detected in the lungs 24 hrs post injection. While this is in line with a previous report tracking the fate of NSCs in stroke [53], Pluchino and colleagues [5] reported that within 24 hrs of injection, primary NSCs had accumulated in the CNS. Similarly, the presence of primary NSCs could be detected in the spleen and lymph nodes within 2 hrs and up to 24 hrs post injection [7]. Using the cuprizone model of demyelination, Crocker et al. [54] also recently reported that ES cell-derived NSCs could traffic to the CNS and reduce demyelination when injected i.v. To allow time for migration into tissues the biodistribution of GS-N cells was assessed at a later time point during the chronic stage of disease, however GS-N cells were not detected in the CNS or in secondary lymphoid tissues. Although we did detect the presence of GFP^+^ cells in the kidney, this was likely to be due to the removal of cellular debris rather than the directed migration of the transplanted cells. The lack of a therapeutic effect of 46C-NS cells supports recent findings by Reekmans et al. [26], in which bioluminescent-based cell tracking studies revealed that adherently expanded NSCs were retained in the lungs following i.v. administration and demonstrated no clinical benefit on the course of EAE. Likewise, it is possible that in the present study, i.v. injected NSCs may have accumulated in the lungs of EAE mice and undergone apoptosis.

We also tested, for the first time, the therapeutic effect of i.p. delivered NSCs as a means of targeting immune responses in the periphery. The reduced pathogenic potential of splenic T-cells in mice receiving high numbers of i.p. injected GS-N cells or 46C-NS cells, as observed in re-call proliferation and cytokine secretion assays, led to some neuroprotective effects within the CNS. In spite of this, we observed no significant impact of 46C-NS cell transplantation on the clinical course of the disease. Moreover, significant amelioration of disease in GS-N cell-treated mice was only observed during the chronic stage of disease. Our results suggest that NSCs may not have been capable of engrafting within peripheral lymphoid organs and mediating long-term clinical recovery when delivered by an i.p. route. Alternatively, the immunosuppressive effects exerted by transplanted NSCs at this time in the disease course, when autoreactive immune cells have already entered the CNS, may not have been sufficient to overcome ongoing neuroinflammatory and degenerative processes. The microenvironment plays an important role in providing extracellular cues that determine the fate and survival capacity of stem cells. Thus, the death of transplanted cells shortly after injection [55], [56] may have been a further contributing factor to the limited therapeutic efficacy of i.p. delivered NSCs, despite the high dose administered. It is also noted that 46C-NS cells and GS-N cells exhibited no impact on serum autoantibody levels. Given differences in the pathogenesis of rMOG-induced EAE versus EAE induced by the encephalitogenic MOG_35–55_ peptide, particularly with regard to the role of B-cells [24], [25], further examination of the immunoregulatory properties of NSCs is required.

In addition to the ability to modulate immune responses, the homing efficiency of systemically transplanted NSCs to sites of inflammation and tissue injury appears to be critically important in this model of CNS autoimmunity. Cellular trans-endothelial migration is orchestrated by a complex group of adhesion molecules, chemokines and their receptors, which permits tissue-specific migration of particular cell types [57]. Flow cytometric analysis revealed that GS-N cells lacked cell surface expression of a number of key homing molecules, including CD44 and integrin-α4. Accumulating evidence indicates that lymphocyte homing to the CNS requires CD44 and integrin-α4 for initial rolling and firm adhesion of lymphocytes to inflamed endothelium [57]. Notably, blocking antibodies against CD44 and integrin-α4 have been shown to inhibit lymphocyte recruitment into the CNS during EAE [58]. Antibodies directed against integrin-α4 were also found to decrease recruitment of NSCs to the brain and spinal cord following systemic delivery [5]. Rampon et al. [59] further demonstrated that the interaction between CD44 and hyaluronic acid also plays a crucial role in the migration of NSCs across brain endothelium under *in vitro* inflammatory conditions. While 46C-NS cells and SVZ-NSCs were found to express CD44 and integrin-α4, all three NSC types used in this study lacked expression of CXCR4. Chemokine receptor signaling pathways provide important cues to direct the migration of cells during tissue injury and inflammation [57]. Specifically, the CXCR4/SDF-1 signaling pathway is known to have an essential role during brain development [60] and has been implicated in the recruitment of lymphocytes and NSCs to sites of CNS pathology [27], [28], [61]. While surface expression of CXCR4 has been previously reported on primary NSCs [5], [61], the lack of CXCR4 expression observed here may be the consequence of *in vitro* cell culture, as demonstrated during *ex vivo* expansion of mesenchymal stem cells [62].

In summary, we demonstrate that the NSCs used in this study, either derived by neural differentiation of ES cells or isolated from the SVZ of adult mice, did not have a major beneficial impact in an EAE model of chronic progressive disease induced by rMOG. Although the bystander immunosuppressive and immunomodulatory properties of NSCs are important, our results suggest that the capacity for homing to inflammatory sites also appears to be a key factor that influences the efficacy of NSCs in this model and should be investigated further. Differences in the therapeutic potential of ES cell-derived NSCs and primary NSCs are not unexpected, given that gene expression analysis has previously demonstrated differences between ES cell and fetal tissue-derived NSCs [63]. Furthermore, extrinsic signals resulting from manipulation of the cell culture environment may have a major impact on the plasticity and function of NSCs [35]. Indeed, it is possible that NSCs derived by other protocols [4], [7], may demonstrate an enhanced beneficial effect in rMOG-EAE. A more extensive comparative analysis of the expression and function of cell surface homing molecules, and how the migratory potential of NSCs may be influenced by the culture environment, was beyond the scope of this study. However, understanding the mechanisms controlling NSC trafficking in neuroinflammatory diseases such as MS is important and may enable these cells to be manipulated as means of improving their homing efficiency, for instance by altering extracellular matrix components during neural induction in order to modulate integrin expression [64] or by enforcing the expression of relevant homing, as demonstrated by *ex vivo* modification of CD44 isoforms on MSCs to improve trafficking to bone [65]. A careful examination of the trafficking potential of NSCs should therefore be performed in parallel with the development of protocols for neural differentiation and expansion of NSCs in order for their full regenerative potential to be exploited.

## Materials and Methods

### Cell culture

46C-NS cells obtained from StemCells Inc (Palo Alto, CA, USA) were generated by neural conversion of 46C ES cells and cultured as previously described [12], [13]. For astrocytic differentiation, 46C-NS cells were seeded in 4-well plates at 1×10^5^ cells/cm^2^ in RHB-A medium (StemCells Inc) supplemented with 1% FBS (Invitrogen, Carlsbad, CA, USA) for one week. For neuronal differentiation, 46C-NS cells were seeded in poly-L-ornithine (Sigma, St Louis, MO, USA)/laminin (Invitrogen) treated wells at 5×10^4^ cells/cm^2^ in RHB-A medium with bFGF (Chemicon, Temecula, CA, USA) alone for the first 6 days and then withdrawing bFGF for the final 8 days of culture. The W9.5 mouse ES cell line [66] used to generate GS-N cells was kindly provided by Professor Stanley. Briefly, ES cells were cultured on mitomycin c-inactivated MEFs on 0.1% gelatin (Sigma) coated 6-well plates and maintained in DMEM supplemented with 15% FBS, 50 U/ml penicillin, 50 µg/ml streptomycin, 2 mM L-glutamine, 1% non-essential amino acids (all from Invitrogen), 1000 U/ml leukemia inhibitory factor (Chemicon) and 0.1 mM 2-mercaptoethanol (Sigma). For EB formation, 250–500 ES cells were added to non-adherent 96-well plates (Costar, Corning, Lowell, MA, USA) in chemically defined medium [19], supplemented with 10 ng/ml bFGF (Chemicon). Medium was refreshed on day 4 and then on day 7. EBs were transferred and cultured for a further 4 days in adherent 96-well plates coated with 0.1% gelatin/4 µg/ml laminin to generate neurospheres. In parallel, EBs were dissociated mechanically and seeded at 1×10^5^ cells/cm^2^ on 10 µg/ml poly-L-ornithine/4 µg/ml laminin coated flasks in neural basal medium (Invitrogen) containing 1% B27, 1% N2 supplement, 2 mM L-glutamine, 1% ITS-G, 50 U/ml penicillin and 50 µg/ml streptomycin (all from Invitrogen) and 10 ng/ml bFGF for 4–6 days. Dissociated cells were termed GS-N cells and were used for *in vitro* and *in vivo* studies. For neuronal differentiation of GS-N cells, bFGF was withdrawn from dissociated day 7 EBs for 14 days. For astrocytic differentiation, dissociated day 7 EBs were seeded onto 12-well plates at 2×10^5^ cells/cm^2^ in neural basal medium supplemented with 2% FBS for 7 days. SVZ-NSCs derived from the brains of adult OG2 Bl6 homozygous mice were cultured as described [67].

### Immunocytochemistry

Cells were fixed with 4% paraformaldehyde for 10 min, washed in PBS and blocked for 2 hr in PBS containing 10% normal goat serum, 1% bovine serum albumin, 0.02% sodium azide (Sigma). Cells were incubated with primary antibody overnight at 4°C and then with the appropriate secondary conjugate. Following antibody staining, 4′, 6-diamidino-2-pheylindole (Invitrogen) was used for nuclear counterstaining. The following primary antibodies were used: mouse anti-nestin monoclonal antibody (1/100), rabbit anti-GFAP polyclonal antibody (1/1000), rabbit anti-MAP2 polyclonal antibody (1/800) (all from Chemicon), mouse anti-βIII-tubulin monoclonal antibody (1/800; Covance, Princeton, NJ, USA), mouse anti-3CB2 monoclonal antibody (1/80) and mouse anti-RC2 monoclonal antibody (1/80) (both from DSHB, Iowa City, IA, USA). Mouse anti-A2B5 monoclonal antibody (1/20) was produced by hybridomas. Secondary antibodies were Alexa fluor 568-conjugated goat anti-mouse IgG (1/800), Alexa fluor 488-conjugated goat anti-rabbit IgG (1/800) (both from Invitrogen) and rhodamine conjugated goat anti-mouse IgM (Chemicon). Fluorescence images were acquired using an Olympus Provis AX70 microscope.

### Gene expression analysis

Total RNA was extracted from 1×10^6^ cultured cells using a High Pure RNA Isolation Kit (Roche) as per the manufacturer's instructions. cDNA was prepared from 0.5 µg of total RNA using SuperScript III First-Strand Synthesis System RT-PCR Kit and oligo (dT) primers (Invitrogen). Quantitative real time PCR experiments were performed using the Light Cycler 480 Real Time PCR System (Roche Applied Science) with Platinum SYBR Green qPCR superMix-UDG (Invitrogen). The thermocycling protocol included an initial cycle at 95°C for 2 min, followed by 40 cycles of PCR at either 95°C for 15 s, 50°C for 30 s and 68°C for 30 s for albumin or 95°C for 15 s, 54°C or 60°C for 15 s and 68°C for 20 s for all other genes. Each sample was run in experimental triplicate. The samples were adjusted to the quantitative expression of β-actin from the same samples. Embryonic heart and Activin A (ActA) treated EB cDNA were used as positive controls. PCR for CNPase was performed using Platinum *Taq* DNA Polymerase High Fidelity (Invitrogen) with 1 µl cDNA under the following cycling conditions: 94°C for 2 min and then 32 cycles of 94°C for 30 sec, 55°C for 30 sec and 68°C for 30 sec followed by 68°C for 10 min. The PCR products were resolved by electrophoresis in 2% agarose gels and the bands were visualized with ethidium bromide under UV light. Primer pairs are shown in Table 2.

**Table 2:** Primer sequences used for gene expression analysis.

Target	Forward	Reverse	Product size
Oct4	5′-CGTTCTCTTTGGAAAGGTGTTC-3′	5′-GAACCATACTCGAACCACATCC-3′	319 bp
Nestin	5′-CCAGAGCTGGACTGGAACTC-3′	5′-ACCTGCCTCTTTTGGTTCCT-3′	161 bp
βIII-tubulin	5′-TCAGCGATGAGCACGGCATA-3′	5′-CACTCTTTCCGCACGACATC-3′	301 bp
GFAP	5′-ACCAAATCCGTGTCAGAAGG-3′	5′-CAGAAGGAAGGGAAGTGCTG-3′	231 bp
MAP2	5′-AGCCGCAACGCCAATGGATT-3′	5′-TTTGTTCTGAGGCTGGCGAT-3′	313 bp
CNPase	5′-CCAAATTCTGTGACTACGGG-3′	5′-GGTTTGCCCTTCCCATAGTA-3′	448 bp
Nkx2-5	5′-TCAAGCCCGAGGCCTACTCTGG-3′	5′-TGGTCTCTCGGCGCCATCCG-3′	248 bp
Tbx5	5′-GGAAAGATGAGGAATGTTCCAG-3′	5′-GTGTTACAGCTGATGTCCTCCA-3′	223 bp
Myh6	5′-ACGCCTAGAGGCCCAGACCC-3′	5′-CCGGCTCGTGCAGGAAGGTC-3′	233 bp
Albumin	5′-GCTACGGCACAGTGCTTG-3′	5′-CAGGATTGCAGACAGATAGTC-3′	266 bp
Brachyury	5′-CATGTACTCTTTCTTGCTGG-3′	5′-GGTCTCGGGAAAGCAGTGGC-3′	313 bp
β-actin	5′-CTGGCCGGGACCTGACAGACTACC-3′	5′-ATCGGAACCGCTCGTTGCCAATAG-3′	228 bp

### Electrophysiology

Electrophysiological recordings were made using standard whole-cell patch-clamp techniques and an Axopatch 200 amplifier (Axon Instruments Inc, CA, USA). The coverslips from four separate experiments containing cells were placed in a recording chamber (Warner Instruments, Hamden, CT, USA) and continuously superfused with physiological saline solution containing: NaCl 137 mM, NaHCO_3_ 4 mM, NaH_2_PO_4_ 0.3 mM, KCl 5.4 mM, KH_2_PO_4_ 0.44 mM, MgCl_2_ 0.5 mM, MgSO_4_ 0.4 mM, glucose 5.6 mM, HEPES 10 mM, CaCl_2_ 1.3 mM, at pH 7.4. Patch electrodes were filled with solution containing; KCl 135 mM, MgCl_2_ 1.2 mM, ATP 1 mM, EGTA 1 mM, HEPES 10 mM, pH 7.2. Data were digitized at 5–20 kHz with a 1 kHz low-pass filter, stored on computer and analyzed using Clampfit 10 (Axon Instruments). Depolarizing current steps were used to evoke action potentials. TTX (3 µm) was used to block voltage gated Na^+^ channels. All chemicals were purchased from Sigma.

### Cell surface expression analysis

Phenotypic analysis by flow cytometry was performed by staining 0.5×10^6^ cells with 30 µl of appropriately diluted primary antibody for 20 min at 4°C. Where applicable, cells were stained with 30 µl of streptavidin Alexa-fluor 647-conjugated secondary antibody (Invitrogen). The relevant isotype control antibody was used as a negative control. Cells were analyzed using a FACSCanto II flow cytometer (BD) and data analyzed using Gatelogic software (Inivai Technologies, Mentone, Victoria, Australia). Primary antibodies were: CD29-FITC, CD44-APC, CD49b-APC, CD49d-biotin, CD49e-biotin, CD49f-PE, CXCR4-FITC (all from BD).

### Recombinant MOG Purification

The extracellular domain of mouse MOG (amino acid residues 1–117 of the mature protein) (rMOG) was produced in the *Escherichia coli* strain DH5alpha using the pQE9 expression vector (QIAGEN) to incorporate an amino-terminal histidine tag as per the manufacturer's instructions. A clarified bacterial lysate containing rMOG was loaded onto a Ni-NTA Superflow column (QIAGEN) under denaturing conditions (6M Guanidine-HCl, 20 mM Tris, 500 mM NaCl, 5 mM Imidazole, pH 8.0) as per the manufacturer's instructions using a BioLogic LP chromatography system (BioRad). Bound protein was washed sequentially with Buffer A (8M Urea, 100 mM NaH_2_PO_4_, 10 mM Tris, pH 8.0), Buffer A (at pH 6.3), Buffer A (at pH 8.0) and finally Buffer B (10 mM Tris-Cl, pH 8). To remove endotoxin the protein was washed by alternating between Buffer C (60% iso-propanol/10 mM Tris-Cl, pH 8) and Buffer B twice each. The protein was then washed in Buffer A again. Refolding of the bound protein was carried out by applying a linear gradient of Buffer A containing 14 mM 2-mercaptoethanol (100%–0%) vs. Buffer D (100 mM NaH_2_PO_4_, 10 mM Tris, pH 8.0, 1 mM reduced glutathione, 0.1 mM oxidised glutathione) (0%–100%). This was followed by a second linear gradient of Buffer D (100%–0%) vs. Buffer E (100 mM NaH_2_PO_4_, 10 mM Tris, pH 8.0) (0%–100%). The bound protein was eluted using Buffer E containing 300 mM Imidazole, and then extensively dialysed over 72 hr against 200 mM NaCl/10 mM Tris, pH 8.0. Protein concentration and purity were estimated using an A_280_nm measurement and SDS-PAGE, respectively. The protein produced was verified as rMOG by western blot analysis using antibodies specific for native MOG.

### EAE induction and cell transplantation

Chronic-progressive EAE was induced in 8–10 week old female C57Bl/6 by subcutaneous injection of 75 µg rMOG emulsified in complete Freund's adjuvant (Sigma) supplemented with 400 µg *Mycobacterium tuberculosis* (Difco, Detroit, MI, USA) into both hind limb flanks. Mice immediately received an intraperitoneal injection of 350 ng *Bordetella pertussis* toxin (Sigma) and again 48 hr later. For cell transplantation, cells were injected either i.v. via the tail vein or into the peritoneal cavity in a volume of 200 µl on days 8, 10 and 12 post immunization. Controls received injections of equal volumes of D-PBS. Mice were monitored daily and clinical scores were assigned according to an arbitrary clinical scale as described [68]. Mice were humanely killed upon reaching a clinical score of 4. All animal experimental procedures were carried out in accordance with the animal ethics guidelines of the National Health and Medical Research Council of Australia and specifically approved by the Monash University School of Biomedical Sciences Animal Ethics Committee (approval number SOBSA/MIS/2006/37).

### *In vivo* tracking of GFP^+^ GS-N cells

Organs from EAE mice injected with GFP^+^ GS-N cells, GFP^+^ fibroblasts or non-injected EAE mice were dissected and genomic DNA extracted using a Gentra Puregene Tissue Kit (QIAGEN, Venlo, Netherlands) according to the manufacturer's instructions. PCR for GFP and the housekeeping gene β-actin was performed with Platinum Pfx DNA Polymerase (Invitrogen) and 100 ng of cDNA under the following cycling conditions: 94°C for 5 min and then 35 cycles of 94°C for 30 sec, 65°C for 30 sec and 68°C for 30 sec followed by 68°C for 10 min. Primer pairs were GFP: 5′-TACGGCAAGCTGACCCTGAAGTTC-3′, 5′-CGTCGTCCTTGAAGAAGATGGTGCG-3′; β-actin: 5′-GGCACCACACCTTCTACAATG-3′, 5′-CGTCGTCCTTGAAGAAGATGGTGCG-3′. The PCR products were resolved by electrophoresis in 2% agarose gels and the bands were visualized with ethidium bromide under UV light.

### Histological analysis of central nervous system tissue

For histological analysis of CNS tissue, the brain and spinal cord were dissected from mice and fixed in 10% formalin (Sigma). Serial sections (5 µm) were cut from paraffin-embedded tissues and stained with H&E, luxol fast blue and Bielschowsky silver impregnation to assess inflammation, demyelination and axonal damage, respectively. Sections were scored blind for semi-quantitative histological analysis of inflammation as follows: 0, no inflammation; 1, cellular infiltrate only in the perivascular areas and meninges; 2, mild cellular infiltrate in parenchyma; 3, moderate cellular infiltrate in parenchyma; 4, severe cellular infiltrate in parenchyma. Demyelination and axonal pathology were assessed by pale staining and scored blind as follows: 0, no demyelination/axonal pathology; 1, mild demyelination/axonal pathology; 2, moderate demyelination/axonal pathology; 3, severe demyelination/axonal pathology.

### T-cell proliferation assays

Spleens were collected from 2D2 mice expressing the transgenic Vα3.2/Vβ11 T-cell receptor specific for MOG_5–55_ peptide (MEVGWYRSPFSRVVHLYRNGK) in the context of 1-A^b^ [22] or rMOG-immunized mice. Single cell suspensions were prepared in complete RPMI medium containing 10% heat-inactivated FBS, 2 mM L-glutamine, 100 U/ml penicillin, 100 µg/ml streptomycin, 50 µM 2-mercaptoethanol and 1 mM sodium pyruvate (Sigma). Following red blood cell lysis, splenocytes were seeded in triplicate in 96-well, flat bottom microtiter plates (Nunc) at a concentration of 2.5×10^5^ cells per well. Splenocytes were cultured in medium alone or in the presence of either 20 µg/ml MOG_35–55_ (GL Biochem, Shanghai, China), 20 µg/ml rMOG, 800 ng/ml ionomycin and 20 pg/ml PMA (both from Sigma), or in wells pre-coated with 10 µg/ml anti-CD3 and 10 µg/ml anti-CD8 antibodies (both from BD). Cells were incubated at 37°C for 72 hr with the addition of 1 µCi/well [^3^H]-thymidine (Perkin Elmer, Waltham, MA, USA) for the last 18 hr of culture. Cells were harvested onto filter mats (Perkin Elmer) and incorporated radioactive nucleic acids counted using a Top Count NXT Scintillation Counter (Packard Biosciences, Meriden, CT, USA). For co-culture experiments, GS-N cells, 46C-NS cells or SVZ-NSCs were added at concentrations ranging from 0.01 to 1.25×10^5^ cells per well prior to the addition of splenocytes.

For analysis of cytokine production, 2.5×10^6^ splenocytes were cultured in 24-well plates for 48 hours in medium alone or in the presence of 20 µg/ml MOG_35–55_, 20 µg/ml rMOG, or in wells pre-coated with anti-CD3 and anti-CD8 antibodies. For co-culture experiments, 1.25×10^6^ GS-N cells, 46C-NS cells or SVZ-NSCs were added. Quantitative analysis of IL-2, IL-17A, IFN-γ and TNF-α was performed using a mouse CBA kit (BD) according to the manufacturer's instructions. Acquisition of events was performed on a BD FACSCanto II flow cytometer and data analyzed and fitted to a 4-parameter logistic equation using the FCAP array software (Soft Flow).

### Anti-MOG antibody determination

Blood was collected at the end point of experiments by cardiac puncture. The total serum antibody response against rMOG was measured by enzyme-linked immunosorbent assay. Briefly, 96-well plates (Nunc, Thermo Fischer Scientific, Roskilde, Denmark) were coated with 100 µl of rMOG (5 µg/ml) in 0.05M carbonate buffer (15 mM Na_2_CO_3_, 35 mM NaHCO_3_, pH 9.6) overnight at 4°C. Wells were blocked with Pierce protein-free blocking medium (Thermo Fischer Scientific) supplemented with 1.5% normal goat serum for 3 hr at room temperature. For total rMOG-specific antibody responses, 100 µl of serum was diluted 1/4000 in PBS. Serum from naïve C57BL/6 mice was used as a negative control and the monoclonal antibody 8-18C5 [69], diluted to 5 µg/ml was used as a positive control. Antibody binding for total rMOG-specific responses was revealed using a horseradish peroxidase-conjugated goat anti-mouse antibody (Sigma) diluted 1/3000. *O*-phenylenediamine (Sigma) was used as a substrate and the reaction performed according to the manufacturer's protocol. Optical density (OD) was measured at 492 nm on a Benchmark Plus Microplate Spectrophotometer (BioRad).

### Statistical analysis

Experimental and control groups were compared using the Student's t test or the Kruskal Wallis with Dunn's post-hoc test. Results are expressed as mean ± SEM. A value of P<0.05 was considered statistically significant.
