# Correction: Comparative Study on the Therapeutic Potential of Neurally Differentiated Stem Cells in a Mouse Model of Multiple Sclerosis

**DOI:** 10.1371/annotation/b1ee2d6e-a1c4-46c9-89a5-c666bbd33d96

**Published:** 2012-06-21

**Authors:** Natalie L. Payne, Guizhi Sun, Daniella Herszfeld, Pollyanna A. Tat-Goh, Paul J. Verma, Helena C. Parkington, Harold A. Coleman, Mary A. Tonta, Christopher Siatskas, Claude C. A. Bernard

The images for figures 5 and 6 were incorrectly switched. The image that appears as Figure 5 should be Figure 6, and the image that appears as Figure 6 should be Figure 5. The figure legends appear in the correct order. 

